# Management of drowning and accidental hypothermia: a national survey of the Major Trauma Centres in England

**DOI:** 10.1186/1757-7241-23-S2-A6

**Published:** 2015-09-11

**Authors:** Louise M Jones, Lauren Shearer, Anthony Hudson

**Affiliations:** 1 St. George's Hospital, London, UK

## Background

Unintentional drowning and accidental hypothermia are important causes of death worldwide, and may occur simultaneously. In the case of severe accidental hypothermia with cardiac arrest, recent guidelines recognised extracorporeal membrane oxygenation (ECMO) and cardiopulmonary bypass as safe and effective rewarming techniques, with survival rates of approximately 50%. We assessed whether the Major Trauma Centres (MTCs) in the UK had protocols for the management of hypothermia and drowning, and whether they had access to ECMO and/or bypass.

## Methods

We conducted a telephone survey during June 2014, asking the on call trauma consultant or clinical director for trauma 4 simple questions. We established whether they had protocols for the management of drowning and/or hypothermia in their Emergency Department, and whether there was access to ECMO and/or bypass. We also contacted a random selection of Air Ambulances for comparison.

## Results

Responses were obtained from 28 of the 30 MTCs (93%). 2 of these had a protocol for drowning (7%), 4 had a protocol for hypothermia (14%), 9 had access to ECMO (32%) and 19 (68%) had access to bypass. Of the 7 Air Ambulances contacted, 6 (86%) had both protocols for drowning and hypothermia and all of those 6 had access to ECMO and bypass.

## Discussion

The relatively infrequent yet serious nature of hypothermia and drowning mean that there has been limited data to establish evidence-based guidelines in managing these conditions. This survey shows that less than 20% of MTC's are currently using protocols, with varying access to ECMO and bypass. The recent evidence in this area highlights the need for a clear and recognised pathway from pre-hospital care to centres that can provide this necessary equipment in order to maximise successful outcomes in these patients.

